# Evolutionary Aspects and Regulation of Tetrapyrrole Biosynthesis in Cyanobacteria under Aerobic and Anaerobic Environments

**DOI:** 10.3390/life5021172

**Published:** 2015-03-30

**Authors:** Yuichi Fujita, Ryoma Tsujimoto, Rina Aoki

**Affiliations:** Graduate School of Bioagricultural Sciences, Nagoya University, Nagoya 464-8601, Japan; E-Mails: ryomacd@agr.nagoya-u.ac.jp (R.T.); euglena6164@yahoo.co.jp (R.A.)

**Keywords:** chlorophyll biosynthesis, analogous enzymes, hypoxia, transcriptional regulator, ChlR, nitrogen fixation

## Abstract

Chlorophyll *a* (Chl) is a light-absorbing tetrapyrrole pigment that is essential for photosynthesis. The molecule is produced from glutamate via a complex biosynthetic pathway comprised of at least 15 enzymatic steps. The first half of the Chl pathway is shared with heme biosynthesis, and the latter half, called the Mg-branch, is specific to Mg-containing Chl *a*. Bilin pigments, such as phycocyanobilin, are additionally produced from heme, so these light-harvesting pigments also share many common biosynthetic steps with Chl biosynthesis. Some of these common steps in the biosynthetic pathways of heme, Chl and bilins require molecular oxygen for catalysis, such as oxygen-dependent coproporphyrinogen III oxidase. Cyanobacteria thrive in diverse environments in terms of oxygen levels. To cope with Chl deficiency caused by low-oxygen conditions, cyanobacteria have developed elaborate mechanisms to maintain Chl production, even under microoxic environments. The use of enzymes specialized for low-oxygen conditions, such as oxygen-independent coproporphyrinogen III oxidase, constitutes part of a mechanism adapted to low-oxygen conditions. Another mechanism adaptive to hypoxic conditions is mediated by the transcriptional regulator ChlR that senses low oxygen and subsequently activates the transcription of genes encoding enzymes that work under low-oxygen tension. In diazotrophic cyanobacteria, this multilayered regulation also contributes in Chl biosynthesis by supporting energy production for nitrogen fixation that also requires low-oxygen conditions. We will also discuss the evolutionary implications of cyanobacterial tetrapyrrole biosynthesis and regulation, because low oxygen-type enzymes also appear to be evolutionarily older than oxygen-dependent enzymes.

## 1. Introduction

Cyanobacteria are prokaryotes that perform oxygenic photosynthesis. Ancient cyanobacteria are believed to be the direct ancestor of chloroplasts in plants obtained by an endosymbiosis event during the early evolution of life [1]. Extant cyanobacteria thrive in very diverse environments, such as in oceans, rivers, lakes, estuaries, hot springs and the surface of rocks on land, some of which are low-oxygen environments [2]. For example, oxygen levels at the bottoms of paddy fields, in mud from estuaries and in eutrophic lakes are very low because of the high respiration of heterotrophic bacteria. In addition, oxygenic photosynthesis can cause oxygen levels to fluctuate during the day [3,4]. For example, the oxygen level just below the surface of microbial mats becomes hyperoxic during the day because of the photosynthetic production of oxygen. In contrast, at night, oxygen is quickly consumed by respiration of heterotrophic bacteria and cyanobacteria, which reduce oxygen levels to near anaerobic levels.

Dioxygen (O_2_) serves not only as a terminal electron acceptor in respiration, but also as a substrate in many oxygenase-catalyzed reactions. Oxygen profoundly contributes to increasing the complexity of metabolic networks in aerobic organisms through various oxygenase reactions [5]. Furthermore, most organisms, including phototrophs, largely change their metabolisms depending on the change in oxygen levels. Purple photosynthetic bacteria switch their growth modes between photosynthesis and respiration in response to the environmental oxygen levels. Extensive studies on the regulatory mechanisms of photosynthesis, including bacteriochlorophyll biosynthesis, have been performed in the purple bacteria, *Rhodobacter capsulatus* and *R. sphaeroides*. Several regulatory proteins, such as RegB-RegA, FNR and CrtJ/PpsR, have been identified as the key factors for the control of the metabolic changes in response to redox changes caused mainly by oxygen in these purple bacteria [6].

Although cyanobacteria grow photosynthetically irrespective of aerobic or anaerobic conditions, these bacteria must contain some regulatory mechanism(s) to cope with hypoxic and anaerobic conditions to survive, because various oxygenase reactions would likely be retarded at night because of limited oxygen. However, knowledge of cyanobacterial adaptation to low-oxygen conditions is limited, because cyanobacteria are regarded as aerobic organisms because of their ability to produce oxygen through photosynthesis. Thus, only nitrogen fixation and hydrogen production have been studied in cyanobacteria as physiological processes under microoxic conditions [7].

We have studied the mechanisms of adaptation to hypoxia by focusing on tetrapyrrole biosynthesis using mainly *Synechocystis* sp. PCC 6803 (*Synechocystis* 6803). This review summarizes recent progress in emerging mechanisms that regulate the biosynthesis of chlorophyll (Chl), heme and bilin in cyanobacteria and highlights the evolution of metabolism in response to the Great Oxidation Event (GOE) that occurred during the late Proterozoic era.

## 2. Coexistence of Aerobic and Anaerobic Enzymes

**Figure 1:**
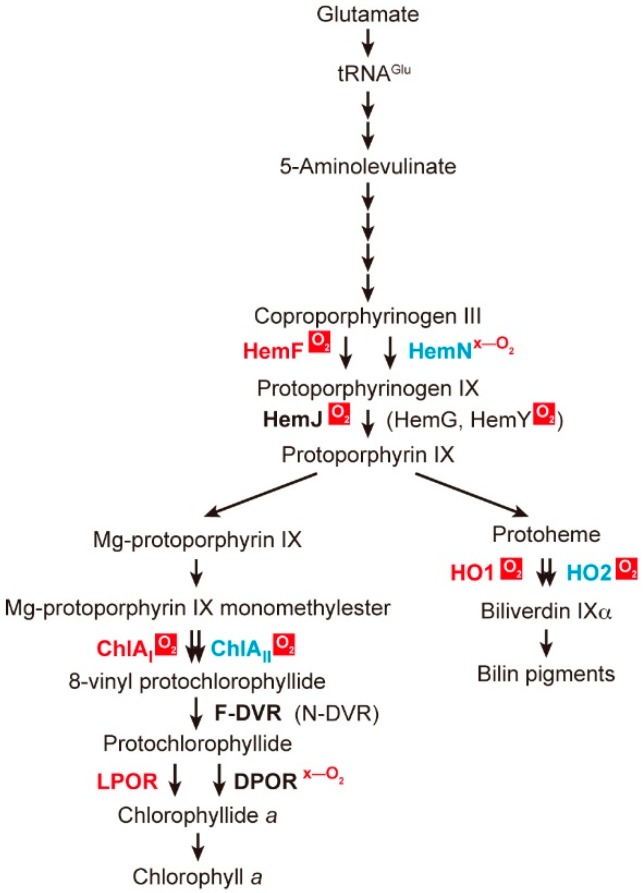
Tetrapyrrole (Chl, heme and bilins) biosynthesis in cyanobacteria. Only the main intermediates and enzyme names mentioned in this review are shown. The complex pathway to produce various bilin pigments after biliverdin IXα is not shown. Steps involving more than two enzymes are shown by two arrows. Two separated arrows indicate that the two enzymes are evolutionarily distinct, and two overlapped arrows indicate that two enzymes are isoforms. Enzymes that have an essential role under aerobic conditions are shown in red. Enzymes that operate mainly under microoxic conditions and the transcription of the genes encoding these upregulated enzymes are shown in blue. Enzymes that require oxygen for catalysis are shown by “O_2_” in the red background, and enzymes that are inactivated by oxygen are shown by red “x-O_2_” symbols.

Tetrapyrrole pigments include Chls, hemes and bilins. These pigments are involved in photosynthesis, respiration and various metabolic reactions in cyanobacteria. The biosynthesis of these pigments includes more than 19 enzymatic steps with a core common pathway from glutamate to protoporphyrin IX (Proto) [8,9,10]. There are at least four enzymatic steps that require oxygen for catalysis (Figure 1). Thus, it would be expected that under oxygen-limited conditions, the production of Chl and bilins becomes retarded by these oxygen-dependent reactions. On the other hand, there are also oxygen-sensitive enzymes that are readily inactivated by oxygen in this biosynthetic pathway (Figure 1). These enzymes may be inactivated under aerobic conditions. Thus, to cope with the circumstances of various oxygen levels, cyanobacteria have developed an elaborate mechanism involving two enzymes that catalyze the same reaction. One enzyme functions under aerobic conditions and the other under anaerobic/microoxic conditions. The expression of genes encoding these enzymes is mainly controlled at the transcriptional level in response to cellular oxygen tension. In the following sections, we will introduce these reactions and review how two enzymes with differing oxygen sensitivity are regulated in cyanobacterial cells.

### 2.1. Coproporphyrinogen III Oxidation

**Figure 2:**
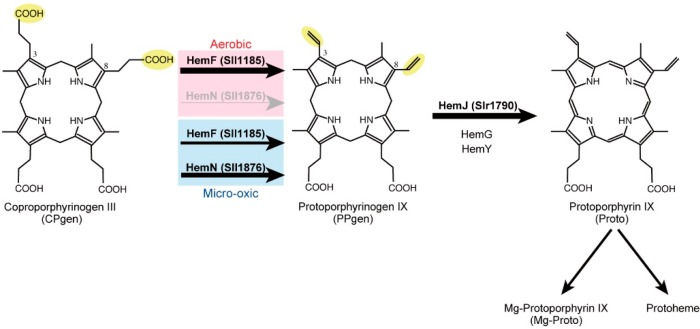
The final sequential reactions to produce protoporphyrin IX: the oxidation steps of CPgen and PPgen in cyanobacteria (*Synechocystis* 6803). In *Synechocystis* 6803, HemF is the sole CPgen oxidase under aerobic conditions, and HemN operates similarly to HemF under microoxic conditions. The produced PPgen is then converted to Proto, the final common precursor for Chl, heme and bilins, by the action of PPgen oxidase. HemJ is the sole PPgen oxidase in *Synechocystis* 6803, as in most cyanobacteria.

Coproporphyrinogen III (CPgen) oxidation involves oxidative decarboxylation of propionate groups at positions C3 and C8 of CPgen to produce protoporphyrinogen IX (PPgen) containing the vinyl groups (Figure 2) [11,12,13]. There are two enzymes, HemF and HemN, which are evolutionarily unrelated, that can undergo this reaction. HemF is a monooxygenase that uses oxygen as an electron acceptor to catalyze this oxidative decarboxylation reaction (EC 1.3.3.3) [14,15]. On the other hand, HemN belongs to the radical *S*-adenosylmethionine (SAM) family [16,17] and, thus, should be called CPgen dehydrogenase (EC 1.3.99.22) [18,19,20,21]. The reaction of HemN proceeds via a 5'-deoxyadenosyl radical generated from SAM. HemN carries a [4Fe-4S] cluster as a cofactor. This iron-sulfur center is disassembled by oxygen, which leads to inactivation in the presence of oxygen. In the genome of *Synechocystis* 6803, there are two genes, *sll1185* and *sll1876*, that encode HemF and HemN, respectively. The enzymatic activities of Sll1185 and Sll1876 were confirmed by reconstitution of reactions with proteins purified after expression in *Escherichia coli* [22].

To investigate the functional differentiation of these two CPgen oxidases, two mutants lacking either *hemF* or *hemN* were isolated in *Synechocystis* 6803, and their photoautotrophic growth was subsequently compared [22]. The *hemF*-lacking mutant (*∆hemF*) does not grow under aerobic conditions, but it can grow under microoxic conditions. This phenotype indicates that HemF is the sole CPgen oxidase under aerobic growth conditions. In contrast, a *hemN*-lacking mutant (*∆hemN*) shows relatively slow growth under microoxic conditions, although it grows as well as the wild-type under aerobic conditions. This indicates that HemF is utilized for PPgen synthesis under aerobic conditions and that HemF and HemN equally contribute to PPgen synthesis under microoxic conditions (Figure 2). One may wonder how HemF operates in such oxygen-limited conditions, because it requires O_2_ as a substrate. Presumably, HemF has an affinity for oxygen that is high enough to bind oxygen even at low oxygen concentrations. This property may allow utilization of endogenous oxygen produced by photosynthesis under microoxic conditions.

In the genome of *Synechocystis* 6803, another *hemN*-like gene, *sll1917*, is also present [22]. The amino acid sequence of Sll1917 shows relatively low similarity (20% and 27% identity) to those of HemN from *Synechocystis* 6803 and *E. coli*, respectively, whereas the cyanobacterial HemN (Sll1876) shows 49% identity to that of *E. coli* HemN. Recombinant Sll1917 protein purified from *E. coli* exhibits an absorption spectrum indicative of the presence of an iron-sulfur cluster. However, no CPgen oxidase activity was detected by assay with purified Sll1917 protein, even though a control reaction using purified Sll1876 protein showed activity under the same anaerobic assay conditions [22]. Interestingly, the *sll1917*-like gene is found in genomes of bacteria devoid of heme biosynthesis, such as *Lactococcus lactis*. Biochemical study of the HemN-like protein from *L. lactis* suggested that this protein has a role in heme trafficking in the cell instead of heme biosynthesis and, therefore, was called HemW, rather than HemN [23].

### 2.2. Protoporphyrinogen IX Oxidation

PPgen produced by CPgen oxidase is subsequently converted to Proto by the action of PPgen oxidase [12]. This reaction involves the removal of six electrons and six protons from PPgen coupled with double-bond isomerization and introduce double bonds between the four pyrrole rings. Proto is the final precursor common to Chl, heme and bilins. Interestingly, three evolutionarily unrelated enzymes, HemG [24], HemY [25] and HemJ [26], are known to catalyze this reaction. HemY and HemG are oxygen-dependent (EC 1.3.3.4) and oxygen-independent (EC 1.3.5.3) enzymes, respectively. The crystal structure of an oxygen-dependent HemY homolog from tobacco mitochondria has been determined [27]. The tobacco HemY PPgen oxidase is a flavoprotein with the structural features suggesting that HemY forms a complex with ferrochelatase, the enzyme that catalyzes the final iron-insertion step of heme biosynthesis. Oxygen-independent HemG is also a flavoprotein, but this protein uses menaquinone rather than oxygen as an electron acceptor [28]. HemJ (Slr1790) was recently found to be a third PPgen oxidase in *Synechocystis* 6803 [26]. The PPgen oxidase assay with a purified Slr1790-like homolog from *R. sphaeroides* in *E. coli* suggests that HemJ is also an oxygen-dependent enzyme, although further biochemical analysis of the cyanobacterial HemJ protein has not yet been performed. Most cyanobacteria, including *Synechocystis* 6803, use HemJ as the sole PPgen oxidase, whereas a few cyanobacteria, such as *Arthrospira platensis*, *Trichodesmium erythraeum* and *Thermosynechococcus elongatus*, have HemY as the sole PPgen oxidase. Two marine cyanobacterial strains, *Prochlorococcus marinus* strains MIT9215 and MIT9515, are unique in that they use only HemG. *Gloeobacter violaceus* PCC 7421 is only one cyanobacterium that has two genes, *hemJ* and *hemY* [29]. The physiological significance of the coexistence of two PPgen oxidases remains unknown. The mosaic distribution of the three enzymes suggests extensive lateral gene transfer during evolution.

### 2.3. Mg-Protoporphyrin IX Monomethyl Ester Cyclization

**Figure 3:**
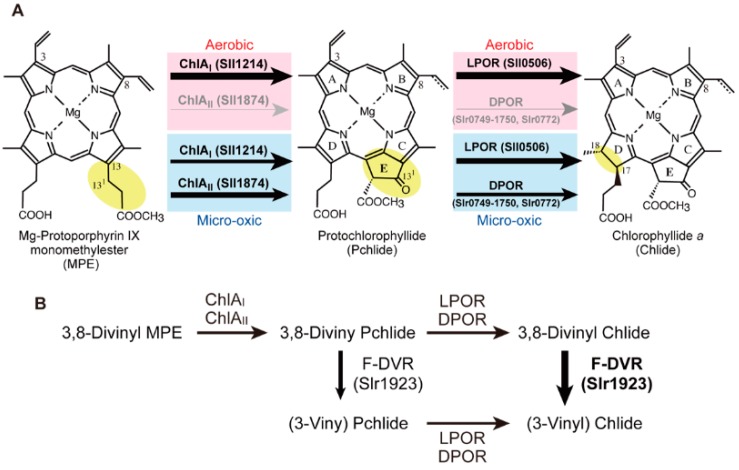
The final three reactions needed to produce chlorophyllide *a* (Chlide), the direct precursor of Chl in cyanobacteria. The thickness of the arrows represents the relative contribution to these reactions. (**A**) The C8-group (vinyl or ethyl) of Pchlide is not specified. In the Pchlide reaction, light intensity and light quality are other important environmental factors in the functional differentiation of light-dependent Pchlide oxidoreductase (LPOR) and the other light-independent (dark-operative) Pchlide oxidoreductase (DPOR), but these factors are not shown in this figure. (**B**) The operation of ferredoxin-dependent 8-vinyl reductase (F-DVR) in cyanobacterial Chl biosynthesis. To emphasize the function of F-DVR, the scheme of the three reactions is simplified without their chemical structures.

The fifth ring “E-ring” (or isopentanone ring) that is structurally specific for Chls is formed by an oxidative cyclization reaction of the propionate at C-13 of Mg-Proto monomethyl ester (MPE) (Figure 3A). This reaction is catalyzed by MPE cyclase that converts MPE to protochlorophyllide (Pchlide) [30]. There are two evolutionarily unrelated MPE cyclases in photosynthetic organisms. One is an oxygen-dependent MPE cyclase [31] with several different names, AcsF [32]/ChlA [33]/Cyc [34]/Chl27 [35]/Crd1/Cth1 [36]/XanL [37] (we denote it *chlA* in this review), and the other is an oxygen-independent MPE cyclase called BchE [38]. Oxygen-dependent MPE cyclase is a monooxygenase that incorporates molecular oxygen to form an oxo group at the C13^1^ position (EC 1.14.13.81) [39,40]. The gene responsible for the oxygen-dependent MPE cyclase has been identified for the first time in the photosynthetic bacterium *Rubrivivax gelatinosus* as *acsF* (aerobic cyclization system Fe-containing subunit), which is involved in oxygen-dependent E-ring formation [32]. In the genome of *Synechocystis* 6803, two genes, *chlA_I_* (*sll1214*) and *chlA_II_* (*sll1874*), were found to show significant similarity to the *acsF* gene. The amino acid sequences of ChlA_I_ and ChlA_II_ show 42% and 39% similarity, respectively, to that of AcsF and show 57% identity to each other. To examine if both genes are involved in the MPE cyclization reaction, two knock-out mutants, *∆chlA_I_* and *∆chlA_II_*, were isolated. The mutant *∆chlA_I_* exhibits a conditional lethal phenotype. *∆chlA_I_* is not able to grow under aerobic conditions, whereas it grows as well as the wild-type under microoxic conditions. When *∆chlA_I_* cells grown under microoxic conditions are incubated under aerobic conditions, a large amount of MPE is accumulated. Although *∆chlA_II_* grows under both conditions, only growth under microoxic conditions is significantly retarded with the accumulation of MPE. These phenotypes suggested that both genes are involved in the MPE cyclization reaction [33]. In *Synechocystis* 6803, ChlA_I_ operates as the sole MPE cyclase (or its subunit) under aerobic conditions, and the contribution of ChlA_II_ to the MPE cyclase reaction is mainly significant under microoxic conditions (Figure 3A).

ChlA is a di-iron monooxygenase with two (DExRH) motifs to bind two irons, which indicates that ChlA is the catalytic subunit of MPE cyclase. MPE cyclase activity has been detected in crude extracts of barley [41], *Chlamydomonas reinhardtii* and *Synechocystis* 6803 [31]. Genetic and biochemical analysis in barley has suggested that at least one protein in soluble fractions and two proteins in insoluble fractions are required for the MPE cyclase reaction [30]. One insoluble protein is ChlA, and the other is probably a protein encoded in the locus of *viridis-k*. In addition, a recent report on the MPE cyclase activity of barley [37] suggested the presence of a third membrane component. However, details of oxygen-dependent MPE cyclase remain enigmatic, because all subunits of MPE cyclase have not yet been identified, and no reconstitution system with purified protein(s) has been established.

NADPH-dependent thioredoxin isoform C and 2-Cys peroxiredoxin were found to significantly stimulate the MPE cyclase activity in barley etioplasts [42]. In addition, Slr1780 was identified as a protein interacting with ChlA_I_ and ChlA_II_ in *Synechocystis* 6803 [43]. The *slr1780* gene is an ortholog of *ycf54* that is a conserved open-reading frame (*ycf*) in chloroplast genomes of algae. Although complete segregation of *∆slr1780* was not successful, a severe phenotype was observed. The Chl content was drastically decreased (about 30% of the wild-type), and MPE was accumulated in the mutant *∆slr1780* [43]. Interaction between ChlA and Ycf54 was confirmed in barley etioplasts [37]. A tobacco Ycf54 ortholog, LCAA, has also been reported [44]. Ycf54 appears to be a new subunit or a protein involved in the assembly process of MPE cyclase.

BchE belongs to the radical SAM family proteins that harbor a [4Fe-4S] cluster and are known to catalyze a wide variety of SAM-dependent reactions [16,17]. Because the [4Fe-4S] clusters of radical SAM family proteins are very vulnerable to oxygen, the activities of these proteins are known to be inhibited by oxygen. The MPE cyclase reaction caused by BchE has been detected in crude extracts, and it was shown to be dependent on vitamin B_12_ (adenosylcobalamin) [38]. However, no reconstitution with purified BchE protein has been reported. As in the case with oxygen-dependent ChlA, establishment of an MPE cyclase reconstitution system with BchE is very important for obtaining detailed biochemical information on E-ring formation, which is a critical key reaction in Chl biosynthesis.

BchE is the sole MPE cyclase in the purple photosynthetic bacterium *R. capsulatus* [45], whereas another purple bacterium, *R. gelatinosus*, uses both ChlA (AcsF) and BchE systems [46]. In the latter species, ChlA and BchE, as well as HemF and HemN are differentially expressed in response to oxygen levels by the oxygen-responding transcriptional factor, FnrL [47], and terminal cytochrome oxidases contribute to lowering of the cellular oxygen tension to aid the expression of *bchE* and *hemN* and their operations [48]. In the filamentous anoxygenic photosynthetic bacterium *Chloroflexus aurantiacus*, both *chlA* and *bchE* genes were found in the genome [49]. The distribution of *chlA* and *bchE* was systemically examined among 53 photosynthetic proteobacterial species. Twenty-one species possessed both *chlA* and *bchE*, and 18 and 14 species possessed only *chlA* and only *bchE*, respectively. Interestingly, although the phylogenetic tree of *chlA* is consistent with that of 16S rRNA, that of *bchE* shows quite complex features, which suggests extensive horizontal gene transfer events for *bchE* [50]. No BchE ortholog has been found in any plants or alga. Analogous to *R. gelatinosus*, cyanobacteria are also expected to possess both oxygen-dependent and oxygen-independent MPE cyclases [30]. In the genome of *Synechocystis* 6803, there are indeed three *bchE* homolog candidates, *slr0905*, *sll1242* and *slr0309*. All three mutants lacking one of these three genes were isolated, but no mutants showed any phenotype, which suggests involvement in the MPE cyclase reaction. Thus, it appears that these genes are not *bchE* orthologs and that the MPE cyclase reaction in *Synechocystis* 6803 is catalyzed only by the oxygen-dependent enzymes, ChlA_I_/ChlA_II_ [33]. However, by extensive BLAST search among cyanobacteria, probable *bchE* orthologs were found in a limited number of cyanobacteria, including *Cyanothece* spp. PCC 7425 and PCC 7822 (Table 1).

**Table 1:** Distribution of *chlR* and aerobic- and anaerobic-/microoxic-type enzymes in cyanobacteria.

F/M ^a^	Species ^b^	*chlR* ^c^	*Cpgen Oxidation*		*MPE Cyclization*			*Heme Cleavage*		Nif ^d^
			*hemF*	*hemN*	*chlA_I_*	*chlA_II_*	*bchE*	*ho1*	*ho2*	
F	*Synechococcus elongatus* PCC 6301	+	+	–	+	–	–	+	–	–
F	*Synechococcus elongatus* PCC 7942	+	+	–	+	–	–	+	–	–
F	*Nostoc punctiforme*	+	+	+ ^¶^	+	+	–	+	+ ^¶^	+
F	*Anabaena* sp. PCC 7120	+	+ ^§,e^	+ ^¶^	+	+ ^§,e^	–	+	+ ^¶^	+
F	*Anabaena variabilis*	+	+	+ ^¶^	+	+	–	+	+ ^¶^	+
M	*Trichodesmium erythraeum*	+ *	+	+	+	–	–	+	–	+
F	*Cyanothece* sp. PCC 7424	+	+	+ ^¶^	+	+ ^¶^	–	+	+ ^¶^	+
F	*Cyanothece* sp. PCC 7822	+	+	+ ^¶^	+	+ ^¶^	+	+	+ ^¶^	+
M	*Cyanothece* sp. ATCC 51142	–	+	+ ^¶^	+	+	–	+	+ ^¶^	+
F	*Cyanothece* sp. PCC 8801	+	+	+ ^¶^	+	+	–	+	+ ^¶^	+
F	*Cyanothece* sp. PCC 8802	+	+	+ ^¶^	+	+	–	+	+ ^¶^	+
F	*Microcystis aeruginosa*	–	+	–	+	–	–	+	–	–
F	*Synechocystis* sp. PCC 6803	+ *	+ ^§^	+ ^¶^	+	+ ^¶^	–	+ ^§^	+ ^¶^	–
F	*Leptolyngbya boryana*	+ *	+	+ ^¶^	+	+ ^¶^	–	+	+ ^¶^	+
M	*Synechococcus* sp. PCC 7002	+ *	+	+ ^¶^	+	+ ^¶^	–	+	+ ^¶^	–
M	*Acaryochloris marina*	+	+	+ ^¶^	+	+ ^¶^	–	+	+ ^¶^	–
F	*Cyanothece* sp. PCC 7425	+	+	+ ^¶^	+	+ ^¶^	+ ^¶^	+	+ ^¶^	+
F	*Thermosynechococcus elongatus* BP-1	–	+	+ ^¶^	+	+ ^¶^	–	+	+ ^¶^	–
F	Cyanobacteria Yellowstone A-Prime	–	+	–	+	–	–	+	–	+
F	Cyanobacteria Yellowstone B-Prime	–	+	–	+	–	–	+	–	+
F	*Gloeobacter violaceus* PCC 7421	+	+	–	+	–	–	+	–	–
M	*Prochlorococcus marinus* MIT 9301	–	+	–	+	–	–	+	–	–
M	*Prochlorococcus marinus* AS9601	–	+	–	+	–	–	+	–	–
M	*Prochlorococcus marinus* MIT 9312	–	+	–	+	–	–	+	–	–
M	*Prochlorococcus marinus* MIT 9515	–	+	–	+	–	–	+	–	–
M	*Prochlorococcus marinus* MED4	–	+	–	+	–	–	+	–	–
M	*Prochlorococcus marinus* NATL2A	–	+	–	–	+	–	+	–	–
M	*Prochlorococcus marinus* NATL1A	–	+	–	–	+	–	+	–	–
M	*Prochlorococcus marinus* SS120	–	+	–	–	+	–	+	–	–
M	*Prochlorococcus marinus* MIT 9313	–	+	–	–	+	–	+	–	–
M	*Prochlorococcus marinus* MIT 9303	–	+	–	+ ^f^		–	–	–	–
M	*Synechococcus* sp. WH 7803	–	+	–	+	–	–	+	–	–
M	*Synechococcus* sp. CC 9311	–	+	–	+	–	–	+	–	–
M	*Synechococcus* sp. WH 8102	+	+	–	+	–	–	+	–	–
M	*Synechococcus* sp. RCC 307	–	+	–	+	–	–	+	–	–
M	*Synechococcus* sp. CC 9902	–	+	–	+	–	–	+	–	–
M	*Synechococcus* sp. CC 9605	–	+	–	+	–	–	+	–	–

Notes: ^a^ Main habitats; F, fresh water; M, marine. ^b^ Thirty-seven species are shown as representative of cyanobacteria. ^c^ The presence and absence of the ortholog are shown by + and –, respectively, based on ortholog search on KEGG (http://www.kegg.jp/). In a search for *chlR* and *bchE*, we performed a BLAST search (http://blast.ncbi.nlm.nih.gov/) to determine their orthologs in cyanobacteria. ^¶^ A gene set forming a gene cluster in the genome. ^§^ A gene set forming another gene cluster in the chromosomal locus other than the gene cluster shown with ^¶^. * These ChlRs have four conserved Cys residues (4Cys-type ChlR). ^d^ The ability of nitrogen fixation is shown by + and –. ^e^ In *Anabaena* PCC 7120, two genes encoding isoforms of HemF and ChlA_II_ are present in the genome, and only one set of *hemF* and *chlA_II_* is located nearby in the opposite directions. ^f^ Because this ChlA homolog shows sequence similarity to both ChlA_I_ and ChlA_II_ by KEGG ortholog search, we regard it as an intermediate ChlA.

### 2.4. Protochlorophyllide Reduction

Pchlide formed by MPE cyclase is subsequently reduced by Pchlide reductase to form chlorophyllide *a* (Chlide) (Figure 3A). Pchlide reduction involves stereo-specific hydrogenation of the C17=C18 double bond with two protons and two electrons. Two evolutionarily unrelated enzymes are known to catalyze conversion of the parental tetrapyrrole ring porphyrin (Pchlide) to chlorin (Chlide). One enzyme is light-dependent Pchlide oxidoreductase (LPOR, EC 1.3.1.33) [51,52], and the other is light-independent (dark-operative) Pchlide oxidoreductase (DPOR, EC 1.3.7.7) [53,54,55].

DPOR consists of three subunits, BchL/ChlL, BchN/ChlN and BchB/ChlB, whose amino acid sequences show significant similarities to those of NifH, NifD and NifK of the nitrogenase subunits, respectively. BchL/ChlL forms a homodimer called L protein, and BchN/ChlN and BchB/ChlB form an α_2_β_2_ heterotetramer complex called NB protein, which are homologs of Fe protein (a NifH dimer) and MoFe protein (an α_2_β_2_ heterotetramer complex of NifD and NifK), of nitrogenase, respectively [54,55,56]. The 3D structures of both L protein and NB protein are very similar to those of nitrogenase Fe protein and MoFe protein, respectively [57,58,59]. The L protein has a role as an ATP-dependent electron donor for the NB protein [60,61]. The NB protein provides the catalytic center for Pchlide reduction [62,63]. Crystal structures of substrate-bound NB proteins have suggested that the spatial arrangement of two proton-donors (BchB-D274 and the C17-propionate of Pchlide) determines the stereo-specificity of this reaction. In addition, DPOR was found to be a radical enzyme that forms Pchlide radicals during catalysis [64], which is a distinct feature from those of nitrogenase. A notable property of DPOR is the high vulnerability of the iron-sulfur clusters of the L protein to disassembly by oxygen, as is the case with nitrogenase [60]. Given that DPOR is distributed among oxygenic photosynthetic organisms, including cyanobacteria, the way DPOR operates in cells that produce oxygen as a byproduct of oxygenic photosynthesis is very interesting [65,66].

In contrast to the enzymatic complexity of DPOR, LPOR is a single polypeptide enzyme belonging to the short-chain dehydrogenase/reductase (SDR) super family [67,68]. Although LPOR is tolerant to oxygen, light is essential for LPOR to promote catalysis of Pchlide to Chlide. The light-dependent property of LPOR is very unique, because photolyase is the only other enzyme that repairs DNA lesions, such as the pyrimidine dimer, and is known to be light-dependent [69]. In the case of LPOR, light is absorbed by the bound Pchlide substrate, which triggers a series of catalytic reactions by LPOR and leads to a hydride transfer from NADPH and a proton transfer from a Tyr residue to complete the stereo-specific reduction of Pchlide [70]. The light dependency of LPOR has enabled a series of extensive biophysical characterizations of the early phases of the catalytic reaction in the pico- to femto-second time frame to be performed [71,72,73]. LPOR is also the sole Pchlide-reduction system present in angiosperms and many eukaryotic algae.

In cyanobacteria, both enzymes coexist and differentially operate under different growth conditions [65,74]. The differences in the enzymatic properties of LPOR and DPOR provide a molecular basis for the differential operation in cyanobacterial cells. In the cyanobacterium *Leptolyngbya boryana*, LPOR is as an essential enzyme under aerobic and high-light conditions [74]. In this species, DPOR operates mainly under low-light or microoxic conditions. The upper limit of the oxygen level that allows DPOR to operate is about 3% in *L. boryana*. This level is coincident with the proposed level of oxygen present during the late Proterozoic era just after GOE [65], which implies that environmental oxidation on the Earth was a selective pressure in the evolution of LPOR from the SDR family as oxygen levels increased above 3%. In the current cyanobacterium *Synechocystis* 6803, PedR, a LuxR-type transcriptional regulator, has a role as a transcriptional activator for the induction of the *chlL*, *chlN* and *chlB* genes of DPOR in response to low-light conditions [75]. The active form of PedR is a homodimer linked by a disulfide bond. This transcription factor is converted to an inactive form upon reduction of the Cys disulfide residues by thioredoxin. The redox state of thioredoxin, in turn, is regulated by the photosynthetic electron flow [76]. Thus, low light is monitored as a redox state of thioredoxin. This regulation is reasonable, because DPOR becomes the sole Pchlide reductase under light-limiting conditions.

Light quality also affects the differentiation of LPOR and DPOR in the filamentous cyanobacterium *Fremyella diplosiphon* [77]. Blue and red light spectral regions are effectively absorbed by the Pchlide bound to LPOR, which effectively promotes catalysis. Because Pchlide poorly absorbs green light, the activity of LPOR is much less under green light growth conditions than those under other light conditions. Under green light conditions, the *chlL-chlN* genes are upregulated, which results in higher DPOR activity to compensate for lower LPOR activity. This behavior could be regarded as a unique “chromatic adaptation” for Pchlide reduction.

### 2.5. 8-Vinyl Reduction

Most Chl pigments have an ethyl group at the C8 position. However, the last precursor common to heme, Proto, has a vinyl group at the C8 position. Thus, the C8-vinyl group must be converted to an ethyl group by an 8-vinyl reductase somewhere along the reactions from Proto to Chlide. There are three evolutionarily unrelated 8-vinyl reductases. One enzyme is NADPH-dependent 8-vinyl reductase (N-DVR, BciA), which is distributed among plants [78], some marine cyanobacteria [78] and some photosynthetic bacteria [79,80]. A second enzyme is ferredoxin-dependent 8-vinyl reductase (F-DVR, BciB), which is mainly found in fresh-water cyanobacteria [81,82] and some photosynthetic bacteria.

N-DVR from *Arabidopsis thaliana* shows high substrate specificity to 3,8-divinyl Chlide, which suggests that the 8-vinyl reduction step occurs mainly at the Chlide stage in *A. thaliana* [83]. In contrast, cyanobacterial F-DVR (*Synechocystis* 6803) shows a broad substrate specificity for the conversion of 3,8-divinyl Pchlide, 3,8-divinyl Chlide and 3,8-divinyl Chl *a* to their ethyl derivatives, which suggests that the 8-vinyl reduction occurs potentially at all three steps: Pchlide, Chlide and Chl *a* [64]. The highest activity was detected for 3,8-divinyl Chlide, which implies that F-DVR catalyzes 8-vinyl reduction at the Chlide step in cyanobacteria [83] (Figure 3B). The activity for 3,8-divinyl Pchlide may become obvious in cells, such as the *chlL* mutant that accumulates Pchlide. In a dark-grown *∆chlL* mutant of *L. boryana*, the accumulated Pchlide is only the 3-vinyl form (Yamamoto and Fujita, unpublished result). In contrast, mutants lacking *chlA_I_* or *chlA_II_* of *Synechocystis* 6803 accumulated 3,8-divinyl MPE [33]. Thus, it is suggested that F-DVR does not catalyze the 8-vinyl of MPE *in vivo* in spite of the broad substrate specificity *in vitro*.

A third 8-vinyl reductase has recently been found in *R. capsulatus*, which is attributed to an alternative activity of chlorophyllide *a* oxidoreductase (COR) in bacteriochlorophyll biosynthesis in the purple bacterium *R. capsulatus* [84]. COR is another nitrogenase-like enzyme that is a very close relative of DPOR. COR catalyzes the reduction of the C7=C8 double bond of Chlide to form 3-vinyl bacteriochlorophyllide *a* [85]. The reconstitution system of COR with purified components clearly indicated that the 8-vinyl group and C7=C8 double bond of 3,8-divinyl Chlide are sequentially reduced by the action of COR [84,86]. This additional activity of COR was confirmed by *in vivo* results [87]. It would be interesting to determine if COR of purple bacteria operates as an 8-vinyl reductase in Chl biosynthesis. However, heterologous expression of COR from *R. sphaeroides* in cyanobacteria causes growth arrest of *Synechocystis* 6803 because of the production of superoxide by COR [88]. The production of superoxide by COR under aerobic conditions might be the main reason that explains why the third 8-vinyl reductase is not distributed among oxygenic photosynthetic organisms including cyanobacteria.

### 2.6. Heme Oxygenase Reaction

**Figure 4:**
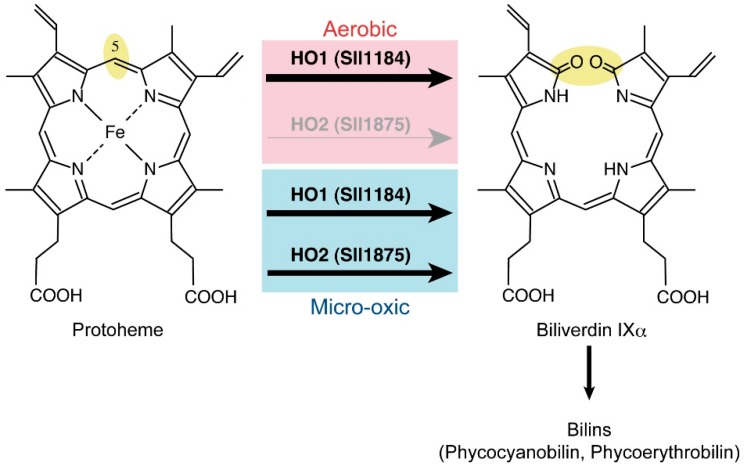
The heme cleavage reaction by heme oxygenases in cyanobacteria. The thickness of the arrows represents the relative contribution to the reactions. Under aerobic conditions, HO1 is the sole HO in *Synechocystis* 6803. HO2 is induced under microoxic conditions to contribute to the HO reaction together with HO1.

Heme oxygenase (HO) catalyzes the oxidative cleavage of heme (protoheme) to form biliverdin IXα, which is the precursor for bilin pigments, such as phycocyanobilin in cyanobacteria and phytochromobilin in plants (Figure 4). HO is an oxygen-dependent enzyme, as indicated by its name. *Synechocystis* 6803 has two homologous genes, *sll1184* (*ho1*) and *sll1875* (*ho2*), which encodes the HO isoforms, HO1 and HO2, respectively [89]. The amino acid sequences of these two HO isoforms show 50% similarity. The activity of both enzymes has been confirmed [90,91,92], and the crystal structures of both isoforms have been reported [93,94].

To determine if two HO isoforms are differentiated in cyanobacterial cells, two targeted mutants, *∆ho1* and *∆ho2*, lacking one of the isoforms were isolated [89]. *∆ho1* showed a conditionally lethal phenotype in which *∆ho1* only grew under microoxic conditions. In contrast, *∆ho2* grew well under both aerobic and microoxic conditions [89,95]. Thus, under aerobic conditions, HO1 has a role as the sole HO essential for photoautotrophic growth. Under microoxic conditions, HO2 acts as an accessory HO. HO2 has the potential catalytic activity to complement HO1, which was confirmed as having the full complement of the lethal phenotype of *∆ho1* by overexpression of *ho2* in *∆ho1* [89]. Comparative biochemical characterization of HO1 and HO2 is being awaited, but these results indicate that HO2 might have higher affinity to oxygen than does HO1, which may enable HO2 to operate under microoxic conditions.

No oxygen-independent heme cleavage enzymes have been found so far, as is the case for evolutionarily unrelated CPgen oxidation and MPE cyclization. Interestingly, HO is found in strict anaerobes, such as *Clostridium tetani* and *C. perfringens* [96]. The probable function of HO in strict anaerobes could involve a role as an oxygen scavenger to maintain anaerobic environments. In addition, the product of HO, biliverdin IXα and its reduced derivative bilirubin are potent antioxidants that may contribute to alleviation of oxidative stress [97].

## 3. Transcriptional Regulator ChlR

**Figure 5:**
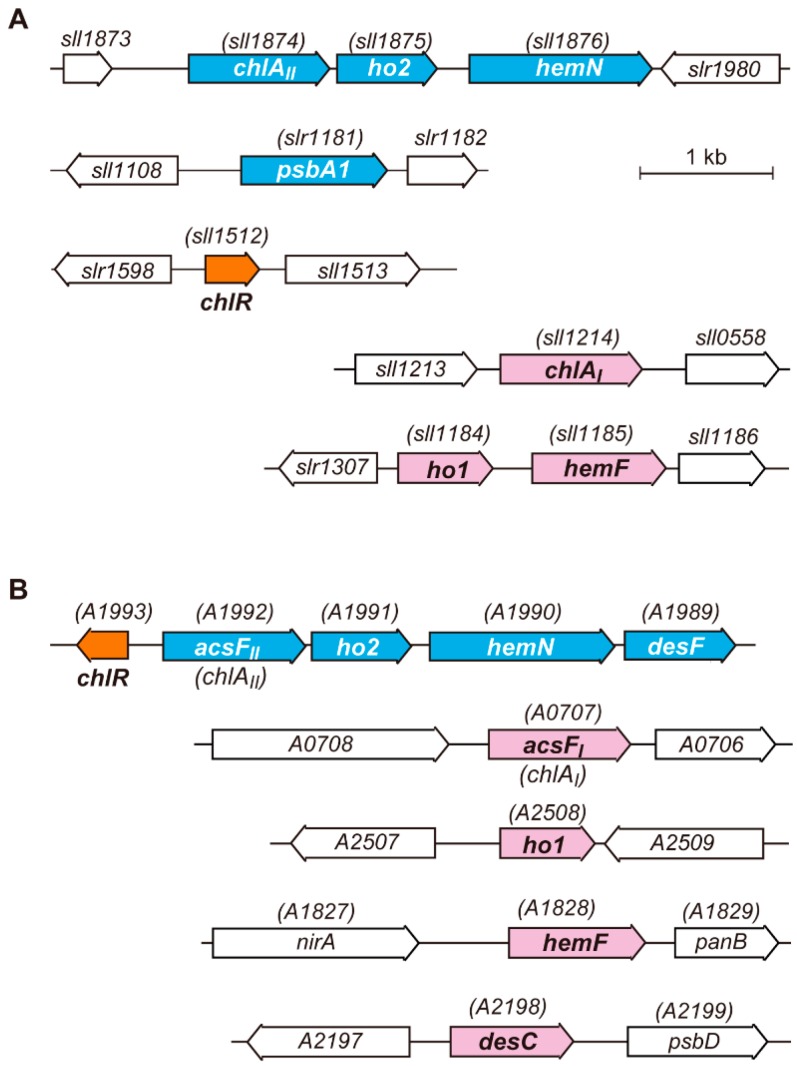
Arrangement of genes for three steps of tetrapyrrole biosynthesis and the *chlR* gene in *Synechocystis* 6803 (**A**) and *Synechococcus* 7002 (**B**). Genes for which expression is regulated by ChlR are shown in blue. The *chlR* is shown in orange. The analogous genes, *chlA_I_*, *ho1*, *hemF* and *desC*, are shown in pink.

Differential expression of two enzymes or two isoforms in three different reactions, CPgen oxidation, MPE cyclization and heme cleavage, is consistent with the oxygen-sensitive and oxygen-requiring properties of individual enzymes. Interestingly, these three genes, *chlA_II_*, *ho2* and *hemN*, which code for anaerobic/microoxic-type enzymes, form a small gene cluster in the genome of *Synechocystis* 6803 (Figure 5A). The expression of these genes is induced under microoxic conditions, whereas their transcription levels are very low under aerobic conditions [22,33,89]. This induction can be interpreted as one of the cyanobacterial hypoxic responses in order to maintain continuous supply of Chl, heme and bilins, even under oxygen-limited conditions. How is the low-oxygen induction of these genes regulated at the transcriptional level? Recently, it has been found that a small protein, ChlR, has a role as a transcriptional activator for the *chlA_II_-ho2-hemN* gene cluster in response to cellular oxygen levels in some cyanobacteria [98,99,100]. In the following sections, we describe the characterization and physiological significance of ChlR for tetrapyrrole biosynthesis under low-oxygen conditions, especially nitrogen fixation requiring anaerobic conditions [100]. The evolutionary implications related to the drastic change in environmental oxygen levels that occurred on the geological scale will also be discussed.

### 3.1. Discovery of the ChlR Regulator

**Figure 6:**
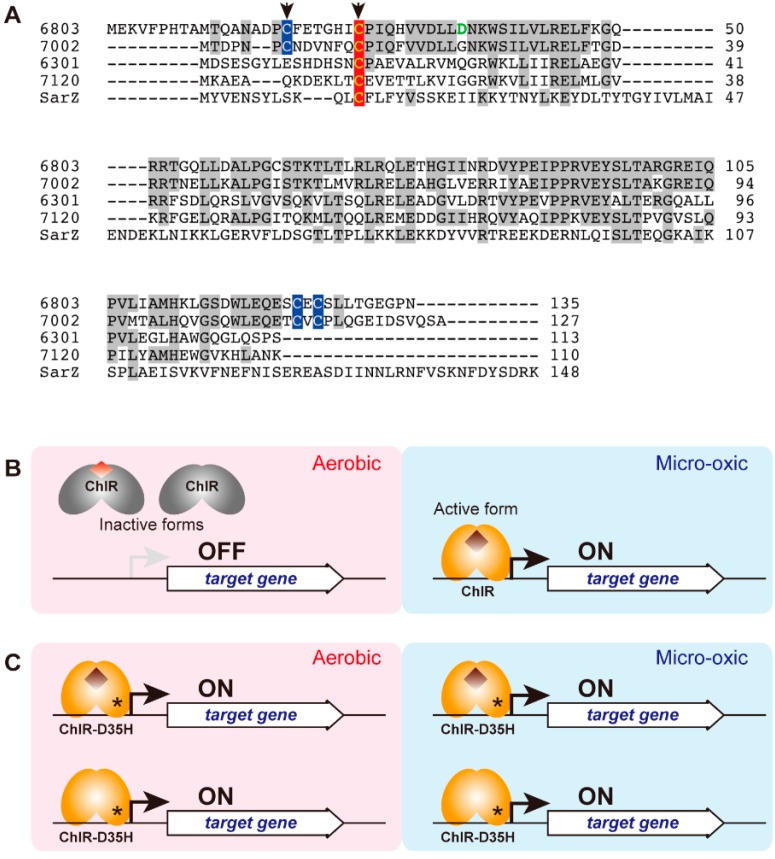
(**A**) Sequence alignment of ChlR and SarZ. The first two ChlRs from *Synechocystis* 6803 (6803) and *Synechococcus* 7002 (7002) are the 4Cys-type, and the other two ChlRs from *S. elongatus* PCC 6301 (6301) and *Anabaena* 7120 (7120) are the 1Cys-type. The Cys residues that conserve all of ChlR are shown in red, and the other three Cys residues conserved in 4Cys-type ChlR are shown in blue. The two Cys residues that may be involved in the iron-sulfur cluster are shown by arrows. The Asp35 residue responsible for the D35H superactivator variant is shown green. SarZ from *Staphylococcus aureus* (SarZ) is shown as a representative of MarR. (**B**,**C**) A working model of ChlR; (**B**) ChlR in the wild-type is inactive under aerobic conditions (left). The inactive form is a dimer with a [2Fe-2S] cluster (colored red brown) or just an apo form without any iron-sulfur clusters. Under microoxic conditions (right), the oxygen level is low enough to keep an oxygen-sensitive [4Fe-4S] cluster (brown diamond), and ChlR can bind the promoter to activate transcription of the target genes (Figure 5). (**C**) A ChlR variant, D35H, is a superactivator that activates the transcription of the target genes even under aerobic conditions. It remains unknown if the ChlR-D35H variant carries the [4Fe-4S] cluster or if it takes the active conformation in the absence of the iron-sulfur cluster.

The *chlR* gene (*sll1512*) was discovered by genome resequencing and genetic analysis of a pseudo-revertant of *∆ho1* in *Synechocystis* 6803 [98]. As mentioned in the previous section, *∆ho1* shows a lethal phenotype under aerobic conditions. A graduate student in our laboratory, Aoki, happened to find a single colony on an agar plate of *∆ho1*. The agar plate was incubated under microoxic conditions followed by exposure to aerobic conditions and later left on a bench for disposal. All *∆ho1* cells were bleached by aerobic incubation, except for one green colony. The green colony was isolated as a pseudo-revertant of *∆ho1* (*∆ho1R*), and it was confirmed that *∆ho1R* grew under aerobic conditions, as well as did wild-type cells. Although the transcripts of *ho2* are detected only in microoxically-grown wild-type cells, the *ho2* transcript was present at high levels in both aerobically- and microoxically-grown *∆ho1R* cells. This aberrant expression of *ho2* appears to complement the HO1 activity in *∆ho1*, which results in normal growth of *∆ho1R* under aerobic conditions. In addition, similar expression profiles were detected for *chlA_II_* and *hemN* in *∆ho1R*. Genome resequencing of *∆ho1R* showed a G-to-C transversion in *sll1512* in the *∆ho1R* strain. This mutation causes a substitution of Asp35 to His in the Sll1512 protein consisting of 135 amino acid residues (Figure 6A). The amino acid sequence of Sll1512 shows significant similarity to those of the multiple antibiotic resistance regulator (MarR) transcriptional regulators present in various prokaryotes, such as HxlR and OhrR from *Bacillus subtilis*. This similarity suggests that Sll1512 has a role as a transcriptional regulator in *Synechocystis* 6803 and, thus, was designated “*chlR*” [98].

### 3.2. Characterization of ChlR

As indicated above, ChlR belongs to the MarR-type transcriptional regulator (MTR) family, which consists of small regulatory proteins typically containing 120–150 amino acid residues. MTRs are widely distributed among prokaryotes and regulate various cellular responses, such as oxidative stress, antibiotic resistance and virulence [101,102]. Sensor (signal input) and regulator (output by transcriptional regulation) domains are fused into a single small polypeptide in MTRs, but they operate as homodimers to bind specific DNA motifs. Most MTRs function as repressors by binding to target DNA motifs to interfere with RNA polymerase binding and/or transcriptional initiation. Binding of a small effector molecule to MTR typically induces a conformational change that releases the molecule from DNA, which results in transcription of the target genes by RNA polymerase. For example, SarZ from *Staphylococcus aureus* is an MTR that controls the expression of genes for organic peroxide resistance [103]. A single Cys13 residue can function as a sensor for organic peroxide by modification to sulfenic acid and mixed disulfide forms (Figure 6A). Although forms with the reduced Cys13 and with Cys13 modified to sulfenic acid bind to the target DNA, further modification of the Cys13 to a mixed disulfide form disrupts DNA binding, which results in the expression of downstream genes.

In contrast to those of most MTRs, the phenotype and transcript profiles of the *∆chlR* mutant suggest that ChlR is a transcriptional activator rather than a repressor. The transcript profiles of the *chlA_II_* gene cluster in the wild-type, *∆ho1*, *∆ho1R* and *∆chlR* suggest that ChlR is inactive under aerobic conditions and is converted to an active form under low-oxygen conditions to bind to the target DNA (upstream of *chlA_II_*), and activates the transcription of the downstream genes, *chlA_II_*, *ho2* and *hemN* (Figure 6B) [98]. The ChlR variant, D35H, in *∆ho1R* is, thus, a superactivator that is constitutively activated even under aerobic growth conditions (Figure 6C). The DNA binding activity of D35H-ChlR was confirmed by the electrophoresis mobility shift assay (EMSA).

ChlR is widely distributed in at least 23 sequenced strains of cyanobacteria, including *Synechococcus* sp. PCC 7002 (*Synechococcus* 7002), *Synechococcus elongatus* PCC 7942, *Anabaena* sp. PCC 7120 (*Anabaena* 7120), *G. violaceus* PCC 7421 and *L. boryana* [99]. An amino acid alignment of multiple ChlR sequences shows that only one Cys residue (Cys25 in *Synechocystis* 6803) is completely conserved (Figure 6A). Thus, this conserved Cys residue might function as a sensor by oxidative modification, as shown in SarZ. Cys25 may be reduced under low-oxygen conditions in which ChlR is in an active form. Under aerobic conditions, Cys25 may be oxidized to a sulfenic acid, which would inactivate DNA binding [98].

An alternative model for sensing of low oxygen by ChlR was proposed by Ludwig *et al.* [99]. In this new model, ChlR may have a [4Fe-4S] cluster that is the sensor for oxygen. EPR and Mössbauer spectroscopy of the chemically-reconstituted ChlR from *Synechococcus* 7002 suggested that a [4Fe-4S] cluster is formed on a ChlR dimer when the cellular oxygen level is low. This form carrying the [4Fe-4S] cluster is presumably the active form. Upon exposure to oxygen, the [4Fe-4S] cluster is altered to a [2Fe-2S] cluster or simply destroyed, which is presumably the inactive form. This cluster conversion in response to the oxygen levels might constitute the oxygen-sensing mechanism underlying the transcriptional regulation of the *chlA_II_* gene cluster (Figure 6B) [99]. To support this model, four Cys residues are required for holding one [4Fe-4S] cluster. Considering that ChlR is a homodimer, two Cys residues are needed per one polypeptide. If limited to 14 cyanobacterial species, four Cys residues are conserved in the ChlR proteins (we call them “4Cys-type” ChlR) (Figure 6A). Two of the four Cys could be involved in chelating one [4Fe-4S] cluster per homodimer. The most probable candidates involved in the [4Fe-4S] cluster are N-terminal Cys18 and Cys25 in ChlR of *Synechocystis* 6803 (Cys7 and Cys14 in ChlR of *Synechococcus* 7002), because the sequence of Cys-X_4-6_-Cys-Pro is quite similar to the well-known iron-sulfur motif Cys-X_2_-Cys-X_2_-Cys-X_3_-Cys-Pro found in [4Fe-4S]-type ferredoxins (Figure 6A) [99]. The use of iron-sulfur clusters to sense oxygen is found in other types of transcriptional regulators, such as FNR and SoxR in *E. coli* [104]. Further biochemical analysis is needed to understand the coordination of the iron-sulfur cluster. Furthermore, it remains unclear how ChlR homologs that have only a single conserved Cys residue operate (Figure 6A). This “1Cys-type” ChlR may use the single Cys to sense oxygen levels or may constitute a paralog that has other physiological functions.

### 3.3. Target Genes of ChlR

There are other genes beyond the three genes involved in tetrapyrrole biosynthesis in which expression is activated by ChlR. One of these is the *psbA1* locus gene in the genome of *Synechocystis* 6803 (Figure 5A). EMSA analysis clearly indicated that the constitutively active D35H-ChlR variant binds to the promoter region of *psbA1* [98]. In *Synechocystis* 6803, there are three *psbA* genes: *psbA1*, *psbA2* and *psbA3*. The *psbA2* and *psbA3* encode the D1 reaction center protein with an identical amino acid sequence. This protein is the sole D1 protein present in photosystem II (PS II) in cells grown under normal aerobic conditions, because no *psbA1* transcript is detected aerobically [105]. The *psbA1* gene encodes a D1 isoform, called the D1' protein, whose amino acid sequence shows 85% identity to that of the normal D1 protein. Because a microoxic expression profile of *psbA1* is similar to those of genes in the *chlA_II_* gene cluster, the D1' protein appears to function in PS II of cells grown under microoxic conditions [106]. Anaerobic environments of natural habitats of cyanobacteria, such as hot springs, rice paddies and estuarine mud, are often accompanied by high levels of hydrogen sulfide. Hydrogen sulfide is an inhibitor of oxygenic photosynthesis, especially at the donor side of the PS II complex [107]. The *psbA1* gene is induced in response to hypoxia, presumably because the D1' protein may function as a special isoform to facilitate rapid turnover of the D1 protein that occurs under anaerobic and hydrogen sulfide-rich growth conditions.

One may expect that some other genes may also be regulated by ChlR. Actually, the *desF* (SYNPCC7002_A1989) gene in *Synechococcus* 7002 has indeed been shown to be regulated by ChlR (Figure 5B) [99]. The *desF* gene encodes a low-oxygen type isoform of fatty acid desaturase DesC, ∆9 desaturase. DesC is an oxygen-dependent oxidoreductase that produces a double bond between C9 and C10 of stearoyl-CoA to convert oleoyl-CoA by oxidation with oxygen. Thus, DesF is probably a DesC isoform with specialized activity under low-oxygen conditions. The amino acid sequence similarity between DesF and DesC is 48%, which is similar to the cases of ChlA_I_/ChlA_II_ and HO1/HO2. In *Synechococcus* 7002, *desF* is located just downstream of *hemN* to form a longer gene cluster, *chlA_II_*(*acsF_II_*)*-ho2-hemN-desF*. In addition, *chlR* is encoded just upstream of *acsF_II_* in the opposite direction (Figure 5B). A *desF*-lacking mutant, *∆desF*, showed a conditionally lethal phenotype under microoxic conditions in contrast to the normal growth under aerobic conditions [99]. This phenotype of *∆desF* suggests an essential role of DesF under microoxic growth conditions. In the genome of *Synechococcus* 7002, the *desC* gene (SYNPCC7002_A2198) is present at a different chromosomal locus (Figure 5B), and DesC is probably the dominant desaturase under aerobic conditions. *∆chlR* and *∆chlR-desF* (lacking the gene cluster, *chlR-acsF_II_-ho2-hemN-desF*) were confirmed as having the same phenotype as that of *∆chlR* in *Synechocystis* 6803 [98,99].

Furthermore, extensive RNAseq analysis of *Synechococcus* 7002 indicated that only four genes, *acsF_II_* (*chlA_II_*), *ho2*, *hemN* and *desF*, are targets of ChlR in *Synechococcus* 7002 [99]. In *Synechocystis* 6803, such transcriptomic analysis has not yet been performed. The transcript levels of 23 genes for Chl biosynthesis (*gltX*, *hemA*, *hemL*, *hemB*, *hemC*, *hemD*, *hemE*, *hemF*, *hemN*, *hemJ*, *chlD*, *chlH*, *chlI*, *gun4*, *chlM*, *chlA_I_*, *chlA_II_*, *cvrA*, *por*, *chlL*, *chlN*, *chlB* and *chlG*) and five genes for photosystems (*psaA*, *psaB*, *psaC*, *psbA2/A3* and *psbO*) were examined by semi-quantitative reverse-transcriptase PCR in wild-type and *∆chlR* cells grown under aerobic and microoxic conditions. Except for *chlA_II_* and *hemN*, the transcript levels of the other 26 genes were almost the same in wild-type and *∆chlR*, irrespective of aerobic and microoxic conditions [108]. Thus, *chlA_II_*, *ho2*, *hemN* and *psbA1* may only be the ChlR-targets in *Synechocystis* 6803.

Finally, another transcriptional regulator involved in the adaptation of low-oxygen conditions is Hik31, a His sensor kinase, in a two-component system in *Synechocystis* 6803 [109]. An extensive microarray analysis suggested that Hik31 negatively regulates the expression of genes for the core proteins of PS I and PS II, ATPase subunits, phycobilisome subunits, some ribosomal proteins and some chaperones under microoxic conditions. Upregulation of the *chlA_II_* gene cluster and *psbA1* under microoxic conditions is not affected in the *∆hik31* mutant. These results suggest that the target genes of Hik31 are not overlapped with those of ChlR. In addition, the *hox* and *flv* genes are upregulated in response to low-oxygen conditions. These genes, thus, are controlled by an unidentified regulatory system other than ChlR and Hik31 [109]. Adaptation mechanisms to low oxygen appear to have independently evolved from a variety of evolutionarily unrelated systems to form a complex regulatory network in extant cyanobacteria.

## 4. Chlorophyll Biosynthesis under Nitrogen Fixing Conditions

Nitrogen fixation is the process of the conversion of atmospheric nitrogen to ammonia, which can be used as an available nitrogen source by many organisms. About half of the characterized cyanobacterial species have the ability to fix nitrogen [2]. Nitrogen-fixing cyanobacteria, therefore, have an important role in the nitrogen cycle in various environments, especially in the ocean surface. The bloom-forming filamentous cyanobacterium *Trichodesmium* and the unicellular cyanobacteria *Cyanothece* and *Crocosphaera watsonii* are recognized as major contributors to the nitrogen balance of oceans.

Nitrogen fixation is catalyzed by the enzyme, nitrogenase. Nitrogenase is comprised of two metalloproteins, Fe protein and MoFe protein [110]: their three metallocenters essential for the catalytic reactions of nitrogenase are extremely vulnerable to oxygen. For example, Fe protein is irreversibly inactivated with a half-life of only about 20 s upon exposure to air [111]. Thus, nitrogen-fixing cyanobacteria evolved various mechanisms to cope with the oxygen inactivation of nitrogenase. In addition, nitrogenase consumes at least 16 ATP molecules and eight electrons per molecule of nitrogen. The reductant for nitrogenase is ferredoxin or flavodoxin in cyanobacteria [7]. Nitrogen-fixing cyanobacteria need to produce large amounts of ATP and reductants to drive nitrogenase. From the perspective of Chl biosynthesis, nitrogen-fixing cyanobacteria should maintain synthesizing Chl and bilin pigments for the production of ATP and reduced ferredoxin by photosynthesis under anaerobic conditions. As mentioned in the previous sections, an oxygen paradox appears in the operation of oxygen-sensitive enzymes, HemN and DPOR, in tetrapyrrole biosynthesis of nitrogen-fixing cyanobacteria.

Many studies on cyanobacterial nitrogen fixation have been performed in *Anabaena* 7120 [112]. Heterocystous cyanobacteria, including *Anabaena* 7120, differentiate heterocysts to provide special environments for the protection of nitrogenase from oxygen. The PS II content is decreased to maintain anaerobiosis in the heterocysts [113] that have special cell walls produced to interfere with the penetration of environmental oxygen into the cell [114], as well as increased respiratory activity to remove oxygen and produce additional ATP needed to support nitrogenase activity [115]. However, it remains unknown how tetrapyrrole biosynthesis is regulated in the anaerobic environments present in nitrogen-fixing cells. Recently, genetic analysis of a non-heterocystous cyanobacterium, *L. boryana*, suggested that ChlR has such a critical role in Chl production to support nitrogenase activity [100]. As is the case for many nitrogen-fixing cyanobacteria, *L. boryana* has the ability to fix nitrogen under microoxic conditions [116]. A 50-kb nitrogen fixation gene cluster was found in the genome of *L. boryana*, and the *chlR* gene is included in the *nif* gene cluster. A *chlR*-lacking mutant does not grow under microoxic nitrogen-fixing growth conditions: in addition, it also accumulates large amounts of Chl precursors, such as MPE, Pchlide and CPgen. The *chlA_II_-ho2-hemN* gene cluster is conserved in another chromosomal locus, and ChlR activates this *chlA_II_* gene cluster by sensing low oxygen, as in *Synechocystis* 6803 and *Synechococcus* 7002. The *chlR* gene is conserved among most nitrogen-fixing cyanobacteria (but not all, because it is not found in *Cyanothece* sp. ATCC 51142); thus, ChlR probably contributes to tetrapyrrole production in nitrogen-fixing cells, including in heterocysts.

## 5. Distribution of ChlR and Aerobic and Anaerobic Enzymes

Table 1 shows the distribution of *chlR* and the aerobic and anaerobic/microoxic types of enzymes for three steps of tetrapyrrole biosynthesis in 37 cyanobacterial representative species. The aerobic-type enzymes, HemF, ChlA_I_ and HO1, are ubiquitously conserved in all species. In contrast, the anaerobic/microoxic-type enzymes, HemN, ChlA_II_, and HO2, are missing in about half of these species. In addition, the anaerobic MPE cyclase, BchE, is found in only two species, *Cyanothece* spp. PCC 7822 and PCC 7425. The *bchE* gene is located just downstream of the *chlA_II_-ho2-hemN* gene cluster in *Cyanothece* sp. PCC 7425. In these *Cyanothece* strains, the *bchE* gene may be regulated by ChlR.

This distribution suggests that aerobic enzymes are the default pathway in current cyanobacteria that are well adapted for growth in aerobic environments. Most species that have anaerobic/microoxic enzymes also carry *chlR*, which suggests that ChlR has a major role as a transcriptional activator for the enzymes, which is similar to its role in *Synechocystis* 6803. However, some strains lack *chlR* despite the presence of the genes encoding for these anaerobic/microoxic enzymes (*Cyanothece* sp. ATCC 51142 and *T. elongatus* BP-1). These species have probably developed another mechanism to regulate these genes. In contrast, four species (*S. elongatus* PCC 6301, *S. elongatus* PCC 7942, *G. violaceus* PCC 7421 and *Synechococcus* sp. WH8102) lack *hemN*, *chlA_II_* and *ho2*, although they carry the *chlR* homolog. Their ChlR proteins may have other unknown functions.

When focusing on different habitats (Table 1), it appears that most fresh-water species have anaerobic/microoxic enzymes, whereas only a few marine species (*T. erythraeum*, *Synechococcus* 7002 and *Acaryochloris marina*) possess them along with *chlR*. This distribution may reflect that the oceanic environments are stably aerobic, whereas the oxygen levels in fresh-water environments, microbial mats and mud, which are often closed systems, are subjected to dynamic fluctuation.

## 6. GOE, Oxygen Crisis and Adaptive Evolution of Tetrapyrrole Biosynthesis

### 6.1. GOE and Oxygen Crisis

**Figure 7:**
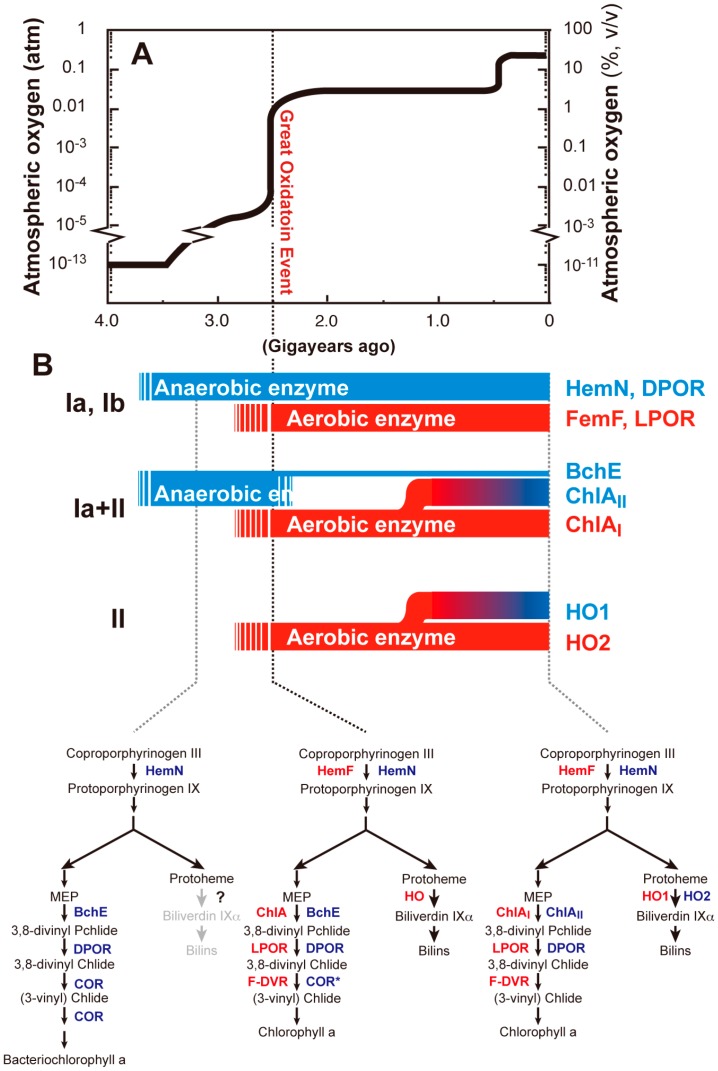
The Great Oxidation Event (GOE) and the evolution of enzymes in tetrapyrrole biosynthesis. (**A**) Proposed change of atmospheric oxygen of the Earth. The atmospheric oxygen level quickly rose about 2.5 gigayears ago, which is known as the GOE [119]. (**B**) Proposed modes of adaptive evolution of enzymes in tetrapyrrole biosynthesis in response to GOE. As shown in Table 2, three different patterns of adaptive evolution of enzymes are shown with classification. Aerobic enzymes may have appeared in response to GOE to compensate for the activities of anaerobic enzymes, except for HO. Adaptive evolution of tetrapyrrole biosynthesis: Only steps after CPgen are shown in the lower part. Aerobic and anaerobic (microoxic) enzymes are shown by red and blue, respectively. A probable biosynthetic pathway established in ancient photosynthetic organisms that produce bacteriochlorophyll *a* (lower left). A probable transient biosynthetic pathway just after GOE in ancient cyanobacteria (middle). COR* is a hypothetical variant with only the 8-vinyl reductase activity to contribute to Chl *a* biosynthesis. The current biosynthetic pathway found in *Synechocystis* 6803 (lower right).

If the continuous supply of oxygen by oxygenic photosynthesis were absent, oxygen in the current atmosphere of the Earth would quickly disappear within a few million years because of its high reactivity [117]. Before the advent of oxygenic photosynthesis in the early evolution of life, the atmospheric environment of the Earth was strictly anaerobic [118]. This means that the early evolution of life had occurred under anaerobic conditions. One of the more critical biogeochemical events in the Proterozoic era was the rapid rise in the oxygen levels, called the GOE, which occurred about 2.4–2.5 gigayears (billion years) ago (Figure 7A) [119]. GOE was caused by the evolution of oxygenic photosynthesis and the widespread massive increase in ancient cyanobacteria that subsequently thrived on Earth. Oxygen is an ideal electron acceptor molecule, as shown by the oxygen-utilizing respiratory chain in extant organisms. Assuming similar efficiencies, energy production in the ancient organisms drastically increased when oxygen reached a level that allowed the evolution of a receptor that could use oxygen as a substrate for respiration. The complexity of metabolic networks in organisms has also remarkably increased with the evolution of large amounts of oxygenases that incorporate oxygen as substrates, which undoubtedly evolved from pre-existing protein families [5]. On the other hand, reactive oxygen species (ROS) derived from reduction and electron spin events involving oxygen (interestingly, singlet oxygen can arise by the interaction of oxygen with light-excited Chl) are known to cause serious damage to organisms that evolved to live in the anaerobic environments [120]. Thus, the increase in environmental oxygen levels caused by GOE must have provided a strong selective survival pressure for all organisms on the Earth.

Ancient cyanobacteria probably spread to light environments on the Earth given the enormous selective advantage they would have had by their ability to convert solar energy into cellular energy. However, their use of water as an electron source for oxygenic photosynthesis assured that they also faced a crisis of cellular oxidative damage as a result of oxygen accumulation that they themselves created. Extant cyanobacteria may have retained ancient metabolic networks that initially evolved in anaerobic environments in which the use of iron-sulfur enzymes was a much more stable option. Furthermore, reduced iron was readily available prior to GOE, which caused a collapse in iron availability when iron became oxidized (ferric iron is much less soluble than is ferrous iron) [121]. Before GOE, primitive cyanobacteria probably synthesized Chl by using anaerobic enzymes, such as HemN, BchE and DPOR, that do not require oxygen. The ubiquitous iron-rich anoxygenic environment prior to GOE would also have provided a stable environment for the [4Fe-4S] clusters that are present in these enzymes. When environmental oxygen levels increased during GOE, the stability of these enzymes would have suffered and resulted in the arrest of Chl biosynthesis and accumulation of Chl precursors that were also themselves reactive with oxygen to form ROS. Additional production of ROS by the oxygen-sensitive Fe-S clusters [121] might have exacerbated the severity of the damage. The “oxygen crisis” was eventually overcome by selective pressure that led to the creation of a series of oxygen-tolerant enzymes to survive. The evolution of the new oxygen-stable enzymes, HemF, ChlA and LPOR, provided substitutes for the more ancient oxygen-labile HemN, BchE and DPOR, respectively.

The rise of oxygen levels during GOE affected not only the enzymes for tetrapyrrole biosynthesis, but also other enzymes in important metabolic networks: this would have led to the creation of numerous oxygen-tolerant enzymes, as well as enzymes that use oxygen as a substrate, such as various oxygenases [122]. Some oxygen-sensitive enzymes might become dispensable and were subsequently substituted by new oxygen-tolerant enzymes. Such global metabolic reconstruction would have generated highly complex metabolic networks, which led to current metabolisms in extant bacterial species [5]. By performing an *in silico* search of metabolic processes, at least 19 reactions, including CPgen oxidation and MPE cyclization, were found as examples of such metabolic replacements [122].

Even though the atmosphere became aerobic, as is the case in the current Earth environment, low-oxygen micro-environments, such as microbial mats and sediments of eutrophic lakes, remain. In addition, cyanobacteria, which rely on respiration for energy production in the dark, also have to withstand anaerobic or microoxic environments during the night, which has occurred every 12 h since the origin of life. Thus, maintaining a set of anaerobic enzymes in these cells probably was an advantage for long-term survival under conditions in which light intensity and oxygen tension fluctuated daily. This might be why many current cyanobacteria still harbor the genes for both aerobic and anaerobic enzymes. ChlR has likely evolved as one of the mechanisms used by these cells to regulate the expression of these genes in response to environmental oxygen levels, as is found in FNR in various facultative anaerobic bacteria [104].

### 6.2. Adaptive Evolution of Tetrapyrrole Biosynthesis

Table 2 and Figure 7B summarize the classification of adaptive evolution modes in the five steps of tetrapyrrole biosynthesis of cyanobacteria. Class I is a mode of coexistence of evolutionarily-unrelated aerobic and anaerobic enzymes in cyanobacteria. Evolutionarily-unrelated enzymes are called “analogous enzymes” [123]. This class is further subdivided into two subclasses, Ia and Ib, by the properties of aerobic enzymes. Subclasses Ia and Ib are aerobic enzymes that are oxygen dependent and oxygen independent, respectively. We also hypothesize that anaerobic enzymes are older enzymes that operated under ancient anaerobic environments and that aerobic enzymes emerged later to compensate for the reduced activities of anaerobic enzymes caused by the rise of oxygen levels during GOE. In the case of CPgen oxidase and Pchlide reductase, these new aerobic enzymes coexisted with the older anaerobic enzymes (Figure 7B). The second class, Class II, is a mode of coexistence of two isoforms that are homologous to each other, such as the aerobic MPE cyclase and HO. In these cases, isoforms specialized to operate under oxygen-limited conditions are induced in response to a decrease in oxygen levels, whereas aerobic forms operate constitutively, irrespective of the oxygen level, and have critical roles under oxygen-rich aerobic conditions. In MPE cyclase, the mode is a mixture of Classes Ia and II. The older anaerobic enzyme, BchE, might have been substituted by the new oxygen-tolerant enzyme ChlA, with ChlA further differentiated into two isoforms in cyanobacteria.

**Table 2:** Modes of adaptive evolution of the biosynthetic pathways of tetrapyrrole in cyanobacteria.

Class ^a^	Reaction	Anaerobic Enzyme ^b^	Aerobic Enzyme ^c^	Extant Cyanobacteria ^d^	Modes of Adaptive Evolution ^e^	Regulator ^f^	Ref.
**Ia**	CPgen oxidation	HemN	HemF *	HemN/HemF	Analogous, coexist	ChlR	[22,98]
**Ib**	Pchlide reduction	DPOR	LPOR ^g^	DPOR/LPOR	Analogous, coexist ^h^	PedR	[65,74,75]
	8-vinyl reduction	COR	N-DVR F-DVR ^i^	F-DVR (N-DVR) ^j^	Analogous, substitution	Unknown	-
**Ia + II**	MPE cyclization	BchE ^k^	ChlA *	ChlA_II_/ChlA_I_^l^	Analogous, substitution ^l^; isoforms ^m^	ChlR	[33,98]
**II**	Heme cleavage	Unknown	HO *	HO2/HO1	Isoforms ^n^	ChlR	[89,98]

Notes: ^a^ Class I, coexistence of analogous anaerobic and aerobic enzymes (Class Ia, the aerobic enzyme is oxygen-dependent; Class Ib, the aerobic enzyme is oxygen-independent); Class II, coexistence of isoforms of aerobic enzymes; ^b^ all anaerobic enzymes in this table are iron-sulfur enzymes; ^c^ oxygen-dependent enzymes are shown by *; ^d^ combination of anaerobic and aerobic enzymes (anaerobic/aerobic) or microoxic and aerobic isoforms (microoxic/aerobic) in extant cyanobacteria exemplified by *Synechocystis* 6803; ^e^ analogous, evolutionarily-unrelated anaerobic and aerobic enzymes are differentially used; substitution, in extant cyanobacteria, only aerobic enzyme(s) are used, whereas anaerobic enzymes no longer exist; isoforms, in extant cyanobacteria, aerobic enzymes are differentiated into two isoforms to adapt to various oxygen levels; ^f^ the cases of *Synechocystis* 6803; ^g^ A gene encoding LPOR was found in some photosynthetic bacteria; the gene for LPOR appears to have been transferred to photosynthetic bacteria from cyanobacteria by lateral gene transfer [124]; ^h^ the genes of DPOR are completely missing in angiosperms, which suggests that DPOR was lost and substituted by LPOR in the evolution from gymnosperms to angiosperms; ^i^ because F-DVR (BciB) from *Chloroherpeton thalassium* was somewhat tolerant to oxygen [125], F-DVR is tentatively regarded as an oxygen-tolerant enzyme in this review; ^j^ some cyanobacteria appear to have N-DVR as the sole DVR; ^k^ BchE appears to be distributed only among photosynthetic bacteria; cyanobacterial orthologs are found only in rare species (Table 1); ^l^ some cyanobacteria, such as *Cyanothece* sp. PCC 7425, have the *bchE* gene in addition to two *chlA* genes; ^m^ some cyanobacteria carry the *chlA* gene as a single gene without any other genes for isoforms; also, plants, such as *A. thaliana*, do not have isoforms of ChlA; ^n^ some cyanobacteria do not have genes for isoforms, and some others have more than three genes for HO isoforms.

The induction of microoxic isoforms (ChlA_II_ and HO2) may have contributed to an increase in enzyme activity as an additive effect under hypoxic conditions, which is a quantitative contribution. Alternatively, these isoforms may have a much higher affinity to oxygen than do those present in aerobic isoforms to accomplish oxygen-dependent reactions under oxygen limitation, which is a qualitative contribution. Biochemical characterizations of these isoforms in future studies are clearly warranted. Oxygen is being generated from PS II under light conditions, even in anaerobic environments. Thus, the retention of microoxic isoforms could be understood as a cellular strategy needed for maximal utilization of endogenously-generated oxygen. The transcriptional regulation of a low-oxygen sensor protein, ChlR, is a purposely-designed molecular mechanism for such an environment.

Given these evolutionary implications, we propose an evolutionary scenario for tetrapyrrole biosynthesis (Figure 7B). Before GOE, the most primitive purple photosynthetic bacteria performed anoxygenic photosynthesis by using bacteriochlorophyll *a* that was produced by a biosynthetic pathway containing the anaerobic enzymes, HemN, BchE, DPOR and COR (Figure 7B, lower left). In this pathway, the C8-vinyl reduction and C7=C8 double-bond reduction were catalyzed by the two catalytic activities of COR. The 8-vinyl reduction may also be catalyzed by F-DVR, or F-DVR may have emerged to complement the loss of COR during the emergence of oxygenic photosynthesis that uses Chl, which does not require COR for bacteriochlorophyll *a* biosynthesis (Figure 7B, lower middle). Upon the rapid rise of the environmental oxygen level during GOE, primitive cyanobacteria had to evolve oxygen-tolerant aerobic enzymes, such as HemF, ChlA and LPOR, to cope with this oxidation crisis (Figure 7B, lower middle). It remains difficult to presume when HO emerged, but probable genes encoding HO are found among anoxygenic photosynthetic bacteria, such as RPA1539, in *Rhodopseudomonas palustris*. It has been reported that bacteriophytochrome holo proteins use biliverdin IXα as the light sensor in *R. palustris*, which gives one function for the presence of HO in anoxygenic bacteria [126]. Alternatively, HO could contribute as a scavenger of trace levels of oxygen to maintain a reduced cellular environment. If the latter is the case, then HO may have evolved very early in GOE as an oxygen-scavenging system. Finally, *bchE* was lost and substituted by *chlA* followed by the generation of isoforms (*ho1* and *chlA_I_*), which led to the currently-established Chl biosynthetic pathway (Figure 7B, lower right), although a small portion of cyanobacterial species (*Cyanothece*) appears still to carry *bchE*. During the final stage, the ChlR regulatory system was incorporated into some cyanobacterial lineages to allow Chl synthesis under low-oxygen conditions, such as those present at night.

## 7. Perspectives and Conclusions

Ancient cyanobacteria are the first organisms to have evolved oxygenic photosynthesis on the Earth. Given that these cells internally generate oxygen as a byproduct of photosynthesis, these cells probably were the first life forms to have faced an oxygen crisis. They are also probably the first lineages to have successfully solved the oxygen crisis. During evolution, many genes encoding primitive anaerobic enzymes appeared to have been lost, but some may have been inherited or retained in current species. Tetrapyrrole biosynthesis is a typical metabolic process that harbors ancient anaerobic enzymes. It is not surprising that anaerobic enzymes still operate in many metabolisms other than tetrapyrrole in cyanobacteria. Indeed, there are 28 genes encoding radical SAM enzymes in the genome of *Synechocystis* 6803. Although some of these genes, such as *hemN*, were found to be functional, most are still hypothetical genes with unknown functions. Given that [4Fe-4S] clusters of radical SAM enzymes are sensitive to oxygen, these enzymes most likely function under microoxic conditions, similar to HemN and DPOR. In this context, cyanobacterial genomes are evolutionary Rosetta Stones that describe the ancient metabolic enzymes and allow a glimpse of the metabolic pathways that may have occurred prior to GOE.

Cyanobacteria and micro-algae are being extensively studied as alternative sources for fossil fuel, such as hydrogen production [127,128,129]. Hydrogenase and nitrogenase are potential enzymes that produce hydrogen, which is a high-energy source for future generations [130,131,132,133]. However, both enzymes have iron-sulfur centers that are very sensitive to oxygen. It will be important to understand how these ancient oxygen-sensitive enzymes are able to operate together with the oxygen produced by photosynthesis. Our knowledge on how cyanobacteria respond to low-oxygen tension is still quite limited, although part of this regulation is just now being revealed. Further extensive analyses using global genomic, transcriptomic and proteomic technologies will help clarify how these cells undertake complex metabolic processes under widely varying concentrations of oxygen. Recent developments of new highly-frequent transposon techniques with *Synechocystis* 6803 appear to be very promising for undertaking additional genetic studies [134]. Spontaneous or random mutations without any tag sequences can be identified by genome resequencing using next generation sequencers [98,135]. In addition, an applicative approach towards the establishment of efficient cyanobacterial systems for biofuel production will shed light on the missing parts of basic approaches.
